# Extracts and Gleanings

**Published:** 1875-04

**Authors:** 


### Extracts and Gleanings.

*Gonorrhœa.*—Dr. W. G. Regester opened the discussion on gonorrhœa. He thought that it is a local disease, and that the local treatment is most important. Injections are valuable, but to make them most effective they should be frequently repeated, and as much depends upon the manner, the physician should introduce them himself. As this was almost impossible in private practice, and it was equally impossible to instruct the patient how to use the syringe properly, he had almost entirely discarded injections, and used soluble bougies, properly medicated with lead, zinc, tannin, bismuth, etc. These bougies are about the size of a No. 9 catheter, and two and a half inches long. Since adopting this mode of treatment, he thought his success was greater and his patients got well sooner.

*Prof. Arnold.* Has Dr. Regester tabulated his cases, and can he tell us the average duration? Some cases get well without trouble, while others continue a long time.

*Dr. Regester.* I have not tabulated my cases, but I think more cases get well quicker than under the old mode.

*Dr. Atkinson.* Bougies are very useful, but they cannot take the place of injections in old cases. The disease is situated in the lacunæ, and deep-seated, and cannot be reached by the bougies. Besides, the injection distends the urethra, and thus is more likely to come in contact with the diseased parts.

*Prof. Arnold.* If the patient can go to bed, I order the penis to be kept immersed in water as cold as he can stand, and for as long a time as he can endure. By persisting in this treatment the majority of cases will get well in three or four days. I think it is a local disease, and cold is as useful as in other local affections.

*Dr. Tiffany.* I have often used ice water injections, and the patient gets well in three or four days, but I think the rest is the important point of this treatment. If you can keep the patient quiet, almost any case can be cured without trouble.

*Dr. Williams.* Can leucorrhœa produce gonorrhœa? If it can, on account of the great frequency with which we meet leucorrhœa, gonorrhœa ought to be very common among married men; but it is not. As far as my observation goes, it is a specific disease, and we sometimes find women with a purulent discharge, and even cancer of the uterus, producing no disease in their husbands.

*Dr. Noel.* I think Ricord gives some cases in which men have caught gonorrhœa from women having these discharges while the husband was perfectly free from disease. He thinks that the husband has become accustomed to it, and thus it fails to affect him.

*Dr. Atkinson.* All pus acts as a poison, and the discharge from purulent ophthalmia will produce this disease.—*Medical and Surgical Reporter.*

*Treatment of Acute and Chronic Bronchitis and Asthma.*—Dr. W. H. Spurgin writes to the *British Med. Jour.* that he has tried iodide of potassium in the treatment of these maladies in over one hundred cases, with almost invariable success; in fact, with such success that patients have expressed themselves by saying, " it has acted like a charm;" others have said that no medicine ever had any real effect upon their complaint before. Iodide of potossium has a marked effect upon the breathing, reducing the frequency of the respirations, perhaps overcoming spasms. Almost after the first dose patients have stated they have felt the medicine touch their complaint.

He usually prescribes it with carbonate of ammonia, and, when the cough is very troublesome, adds tincture of belladonna and ipecacuanha wine.

In one case of very severe broncho-pneumonia he tried iodide of potassium, with tincture of hyoscyamus and ammonia, and the respirations were quickly and astonishingly reduced from forty in a minute to less than half than number.

He adds, in conclusion, that he has purposely given a mixture containing ammonia, belladonna, ipecacuanha wine, spirit of sulphuric ether, etc., without iodide of potassium, without finding much benefit; after which he added iodide of potassium, and found the patient relieved almost at once.

He confidently recommends iodide of potassium as the remedy in these troublesome complaints.—*Druggists' Circular.*

*The Wet Sheet in Scarlatina.*—Dr. Taylor (*Lancet*) thinks that the above "simple, powerful and ready-at-hand auxiliary" in the treatment of scarlatina is not properly appreciated by the profession. An experience of forty years has served to assure him "that this plain or medicated vapor-giving envelope affords the best *external means* for eliminating scarlatinal poison and preventing destructive sequelæ." It promptly suppresses pyrexial heat and itching; produces more or less continuous sleep, with a soft, secretive skin; and enables the digestive organs to accomplish the great desideratum in scarlatina, namely: absorption of highly nutritious food. It may be repeated, on the recurrence of the febrile paroxysms, two, three or four times in twenty-four hours, the patient remaining enveloped from half an hour to an hour. Mothers and nurses who have witnessed its efficacy are most earnest for its repetition. His plan of procedure is to immerse a night-gown, slit up at the front, in hot water (half pint to a pint), pure or medicated with a drachm or two drachms of tincture of capsicum, or in the infusion of three or four pods; or in mustard-water, the clear, supernatant fluid from a tablespoonful of mustard to a pint of water; extending the gown over the feet by means of a towel immersed in the same fluid, both to be well wrung out and suddenly applied, the patient quickly packed in two blankets previously placed on the adjoining sofa or bed; another blanket, or two pillows, or an eider-down quilt, covering all.

The medicated packing is preferable in the incipiency, and at any other time to evoke the rash, and in cases of cerebral oppression, with pale skin, low pulse and delirium.

The auxiliary mode of treatment here defined is by no means intended to exclude the ordinary plan which every practitioner's experience has led him to select and to rely upon; but the author believes that if packing is judiciously incorporated with such reliable treatment, it will be the means of saving many lives that would otherwise be lost, and of diminishing the severity and duraaion of the sequelæ.—*Boston Med. and Surg. Jour.*

*Soothing Application in Herpes Zoster.*—℞. Collodion, ℥j.; morphiæ muriat., gr. viij. M. To be painted over the vesicles without breaking them open.—*Canada Lancet.*

*New Researches on Diabetes.*—We learn that Dr. Pavy has obtained some experimental results which are likely to throw a new light on the subject of diabetes. He has found that the injection of defibrinated arterial blood into the portal system occasions a saccharine state of the urine. In one experiment, the urine after the operation contained fifteen grains of sugar to the fluid ounce, and in others the quantity has amounted to nearly the same. In the counterpart experiment of injecting defibrinated venous blood into the portal system, the urine showed no signs of the presence of sugar. It thus appears that oxygenated blood passing to the liver causes an escape of sugar from the organ, and thence an accumulation in the system, and discharge with the urine. It also appears that, through the medium of the respiration of oxygen, he has succeeded in inducing a sufficiently oxygenated state of the blood to similarly give rise to the production of saccharine urine. He has further found that through the agency of the inhalation of puff-ball smoke an immediate and strongly diabetic state may be induced, and that the effect is accompanied with such a modification of the circulation that the blood flows through the vessels, as is the case after section of the sympathetic, without becoming properly dearterialized. His experiments, he considers, suggest that, in diabetes of the human subject, the blood, in consequence of vaso-muscular paralysis, is allowed to reach the portal vein in an imperfectly dearterialized condition, and thus determines the escape of sugar from the liver. We understand his results are to be brought forward at the Royal Society as soon as its meetings commence. —*Lancet.*

*Remedy for Dysmenorrhœa.*—Dr. Edis recommends in some cases a suppository of half a grain of morphia with one-seventh of a grain of atropine inserted at bed time, in dysmenorrhœa. This, he says, will often allay the most severe pain, and enable the patient to procure sleep, when otherwise she would have passed the night in agony, the stomach itself refusing to absorb anodyne mixtures, rejecting them as soon as swallowed, and thus cutting the patient off from the ordinary means of relief. In other cases of dysmenorrhœa he recommends an enema of water as hot as the patient can conveniently bear, combined or not with half a drachm of laudanum.—*Medical Press and Circular.*

*Eczema of the Ear.*—Dr. Charles S. Turnbull, in a letter from Vienna, Austria, to the *Philadelphia Medical and Surgical Reporter*, says:

In poulticing the ear, as for abscess (furuncle) in the meatus, he lays great stress upon the necessity of making a cone of linen to contain the substances that are used, for they must not come in contact with the membrana tympani. He condemns the indiscriminate instillations of all essential oils and glycerin, on account of its souring. In eczema of the external ear in children, or in women, especially that form so often seen accompanying irregular menstruation, or about the time of the menopause, he uses unguentum glycerinæ for children, and for adults adds five grains of zinci sulph. to the ounce. The salve is not only to be rubbed in, but in every case spread on lint and kept applied for several days, and at the end of that time the parts are not to be washed, but dried, and dusted with amylum, or any toilet powder. For the chronic form use: ℞. Amyli puri, ℥ iv.; pulv. iridis florent, alum. pulvis. aa. ʒ ij. M. until the new epidermis is fully formed. Where there is great thickening due to chronic infiltration of the part with very little transudation of serum, the case being peculiarly obstinate, it is well to excite reaction by rubbing on a little sapon. viridis (a soap containing a large quantity of potash), then applying ung. diachyli, ℞. Emplastri diachyl. liquef., olei. lini coct., aa. ℥ ij.; olei. lavendulæ, gtts. vj. M. spread on pieces of linen, being careful to keep it *well in contact* with the parts day and night, until the new epidermis is fully formed. For chronic eczema and its sequelæ, vesicants behind the ear over the mastoid process; when there is pain in the ear from inflammation of the external auditory canal, or myringitis, etc., the following ointmeut is to be spread upon a pledget of lint and introduced into the meatus at night: ℞. Ung. emoll, plumb. carb., aa. ʒ j; morph. sulph., grs. iij. M.

*Onychia Maligna and Ingrowing Nail Fingers.*—Both of these troubles can be relieved by the local application of powdered nitrate of lead to the inflamed and ulcerated part. But few applications are needed, about once in three or four days. The projecting edge or edges of the nails should first be clipped away.—*Canada Lancet.*

*Psoriasis Treated by the Internal Use of Carbolic Acid.*—Moritz Kolin, of Vienna, has recently published in the *Arch. f. Derm. u. Syph.*, the record of numerous cases of various forms of skin diseases and syphilis, treated by the internal administration of carbolic acid. Cases of psoriasis, pityriasis rubra, prurigo, pruritus cutaneus, and certain non-ulcerative forms of syphilis, improved greatly or or were wholly relieved after the use of this remedy, during a period of from four to seven weeks. It was administered in the form of pills made up with liquorice, each pill containing one grain of carbolic acid. At first, from six to nine pills were given daily, and later from twelve to twenty. He noticed that even after a small amount was taken it could be detected in the urine, though it was never the dark thick urine, smelling of tar, which has been observed after applications of carbolic acid to open wounds. In illustration of this methed, he mentions two cases of psoriasis occurring in his own practice. One was a man twenty-six years of age, who took from four to sixteen grains daily, and at the end of five weeks there was entire disappearance of the disease. The only adjuvant in the treatment was cod-liver oil, which was used as an inunction in the later stages. After the lapse of three months there had been no recurrence of the disease. The other patient was a girl, thirteen years of age, with whom the same treatment was pursued, but without the cod-liver oil, and she recovered entirely of the disease at the end of four weeks. She never took more than four grains a day. At the expiration of four and a half months there had been no relapse.—*Allg. Med. Central-Ztg.*, No. 103.

*A Difficulty in Fœtal Auscultation.*—Dr. J. Braxton Hicks calls attention to a point with regard to the diagnosis of pregnancy and the life of the fœtus, by means of the existence of the fœtal heart-sounds—which he had not unfrequently observed in the course of his practice, but which he does not remember to have seen in print—and summed up his observations as follows: First, that the number of vibrations of the abdominal muscles in a state of half-suspension can be distinctly counted, watch in hand; second, that their number and sound is so like those of a very rapid fœtal heart that they may be mistaken for them.—*Philadelphia Medical Reporter—Medical Examiner.*

*The Elastic Ligature.*--In an introductory lecture, Professor Buchanan, of the University of Glasgow, narrates the following anecdote:

At the present time improvements in surgical manipulation are daily being introduced. Three or four weeks ago, on entering the infirmary at the morning visit, a foreign gentleman presented me a card of introduction from a friend at that time residing in Germany. The writing was not very legible, and I did not notice the name, but asked the stranger to accompany me. Standing beside the bed of a patient who was recovering from a compound depressed fracture of the frontal bone, without having had a bad symptom, I said, "We are indebted in great part to one of your countrymen for our present improved mode of treating such cases. We have been taught almost to abandon the use of the trephine by Stromeyer, of Hanover." "My father-in-law," replied my visitor. "Indeed," I exclaimed, "and you are?" "Esmarch, of Kiel." It so happened that on the morning in question I was to remove a large vascular scrotal tumor. Dr. Esmarch accompanied me to the theatre, and the patient was put under the influence of chloroform.—Turning to Dr. Esmarch, I said, "It is a great pity that your bloodless method is not applicable to such a case, as I shall operate with great anxiety, owing to the exhausted condition of the man and the great vascularity of the tumor." He at once undertook to apply the pressure in a way, he believed, would prevent any hemorrhage. Being provided with a long India-rubber tube, he passed it round the base of the scrotum, across the pubis, and around the loins, and fixed it in front of the abdomen. To my surprise and delight, I removed the great mass by a complicated and tedious dissection, without the loss of any blood except what escaped at the first incision from the gorged veins on the surface. The man was dismissed with the wound quite healed in three weeks, and I have no doubt the rapidity of his recovery was, in great part, owing to his having been spared the loss of blood.—*Medical and Surgical Reporter, Philadelphia.*

*Suppurating Bubo.*—Free incision, and pack the cavity with lint wet in a lotion composed of one drop of sat. sol. of carbolic acid and six drops of sweet oil.

*Dysmenorrhœa.*—Some useful clinical remarks by Dr. Peter, of the Hospital St. Antonio, of Paris, are given in the *Lancet:*

Dr. Peter lays stress on the uselessness of the blister and the effectiveness of the scarificator, and remarks that if he did not use depletion immediately, it was because, in France, and especially in Paris, medical men are prevented from doing so by a fashionable prejudice which he calls "the spectre of anæmia." "Through a wild terror of this spectre," he says, "we allow inflammation to go on and become formidable, for want of abstracting a few drops of blood; and certainly, in this case, few physicians would have consented to an application of the scarificator at the beginning of the disease, and the same would have been accepted only, perhaps, when the increasing gravity of the symptoms would have called for urgent measures." Besides the efficacy of the blood-letting, Dr. Peter called attention, in connection with this case, to other points which should also be mentioned, namely, the diagnostic value of pain in the lower part of the back, and the advisability, for diagnostic purposes, of exploring the abdomen with only one finger. "In uterine affections, especially," he said, "we can thus localize the evil with the greatest precision, and in cases of puerperal affection it is of the highest importance to do so, for it is then necessary to make out whether the uterus and its appendages are the primary seat of the mischief, whether the pelvic peritoneum is involved in consequence, whether itself alone or the whole peritoneum is involved; and upon this important diagnosis, which can be made with only one finger, depend both prognosis and treatment."—*Med. and Surg. Reporter.*

*Treatment of Hydrarthrosis.*—In cases of dropsy of the joints, especially that of the knee, Dr. Bergeret finds that continued application of bags of hot sand answer better than any other kind of treatment. When the acute stage is passed, and whatever may be the cause of the dropsy, he wraps the joint in a thick layer of cotton-wool, and applies to this a sack containing two or three litres of fine and very hot sand. The dropsy disappears in a few days. The sand must be very hot, and the heat may be kept up by means of covering with a blanket. The sand must not be too thick in the bag, so that it may extend easily on the knee, and overhang the hydrarthrosis in every direction.—*Journal de Therapeutique.*

*Cold-Powder.*—We have long been in the habit of using what we call a *cold-powder*, which we have found of great value in breaking up cold when taken in time, and in modifying force when taken late. The prescription is as follows:

Camphor, five parts. Dissolve in ether to the consistence of cream. Then add carbonate of ammonia, four parts; opium-powder, one part. Mix and keep in a tightly-corked bottle.

The dose is, of course, regulated by the opium, and ranges between three and ten or fifteen grains. We have been accustomed to prescribe it for our friends by the finger-nail full, or as much as can be put on the finger-nail.

This powder may be taken in a little water just before retiring, by preference, or at any hour of the day, whenever there is a suspicion of having caught cold. If need be, a moderate dose may be taken several days in succession.

The advantages of this powder are very great.

1. The taste is agreeable, or at least it is not disagreeable. Even the bitterness of the opium is mostly neutralized by the camphor and ammonia. No child objects to this powder.

2. It is singularly and inexplicably efficacious. We believe it to be more efficient than Dover's powder, and incomparably more agreeable. In some cases it produces a gentle perspiration; in others, this especial effect is not observed. It is so easy to take, and so harmless in small doses, that it is well and safe to take it whenever we become badly chilled.

We first called attention to this *cold-powder* in "Our Home Physician," the first edition of which appeared in 1869. From various sources, lay and medical, we hear that it accomplishes all that is here claimed, and we therefore earnestly recommend it to the profession.

Quite a large proportion of nervous diseases, as locomotor ataxia, spinal congestion, neuralgia, progressive muscular atrophy, and-so-forth, are excited or aggravated by taking cold; anything, therefore, that will lay the ax at the root of the tree and remove the cause of those diseases, is worthy our study.—*Archives Electrology and Neuralogy.*—*Missouri Record.*

THREE-FOURTHS of the difficulties and miseries of men come from the fact that most want wealth without earning it, and fame without deserving it.

*The Capacity of Plants to Absorb Arsenic from the Earth.*—At the last annual meeting of the National Academy of Sciences a paper was read by Dr. J. L. Leconte on "The Use of Mineral Poisons for the Protection of Agriculture." There was considerable discussion on the subject of the paper. Paris green, it appears, has been recommended for the destruction of the "Colorado potato bug," and, as a consequence, it was said the demand for the article had become so large that one chemist in Baltimore manufactured 3,000 pounds a day, and druggists in small Western towns ordered it by the ton. It is composed of arsenic and acetate of copper, which are poisonous to both animal and vegetable life. It would not be taken up by the leaves of plants, but would be washed into the soil, which would soon become incapable of supporting vegetation. It was a grave question what chemical changes might take place in the soil that would enable plants to take up these poisons; that such may occur has been recently proved by analyses made by Professor Silliman. The loose way in which this poison was handled, and the absence of all restriction upon its sale, were very alarming. Many deaths had occurred, either through accident or design, from Paris green which had been purchased to kill roaches or other vermin.

The increasing sale and use of strichnia for similar purposes was also alluded to. The general use of aniline colors in candy-making and for coloring syrups and jellies in immitation of the fruits from which they were supposed to be made, was instanced as one of the ways in which the health of the people was daily being injured.

Dr. David Davy, years ago, tested the capacity of plants to take arsenic from the soil. Experiments were made with turnips grown on ground that had been fertilized with superphosphate of lime, in the preparation of which impure sulphuric acid had been used, and arsenic, on analysis, was readily detected.—*Druggists' Circular and Chemical Gazette.*

*Pruritus Vaginæ.*—A writer in the *Lancet* strongly recommends a dilute solution of the muriate of iron in this complaint, and also in pruritus ani. Its use is local, we presume, though it is not clearly stated.

*Rheumatism.*—Dr. T. S. Dowse, in a paper read before the Norwich meeting of the British Medical Association, and published in full in the *Journal*, says that in the treatment of rheumatic fever the first thing to do is to eliminate the acid products of the diseased state; and the next, to relieve pain. To bring this about, he has for the last three years been in the habit of packing most of his patients in a wet blanket, and then rolling them up in dry blankets, so as to promote profuse sweating, and also increase the temperature. Finding this rough method gave good results, he at once adopted a systematic mode of procedure, which he thus describes: The bed is covered with India-rubber sheeting; over this is laid a blanket which has been wrung out of hot water. The patient is then enveloped in the blanket, and covered with six folds of dry blanketing. By this the temperature is raised, and profuse sweating results; the former, if need be, is assisted by the administration of brandy in half-ounce or ounce doses every hour, and the latter by giving freely warm milk and water. If the temperature exceed 102° F., then the stimulant is unnecessary.

Dr. Dowse continues the treatment for three days. He finds that after the third pack the pain has completely subsided, and the sour taste usually disappears.

We may remark that this method of treatment is by no means new.—*The Doctor.*

*On Iced Clysters in Dysentery.*—Dr. Wenzel (*Annales de la Société de Médecine d' Anvers* and *L' Independente*) having had occasion to treat a great number of cases of dysentery, has found the best remedy to consist in the injection of ice-water into the rectum. The first case he treated in this way was one of severe dysentery. There were intense fever, abdominal pains, excruciating tenesmus, and profuse sanguineous evacuations. To check the hemorrhage, injections of ice-water were ordered every two hours, which not only caused the sanguineous evacuations to cease, but also removed the tenesmus, enteric pains and fever. The beneficial effect of these injections was so evident that the patient urgently demanded their repetition whenever the pains threatened to reappear. Dr. Wenzel considers this treatment more satisfactory than any other in acute cases, although in chronic cases it can only be expected to afford temporary relief.—*London Medical Record.*

*New Treatment of Boils.*—Dr. Madison Marsh, of Port Hudson, La., (*Medical and Surgical Reporter*,) says that boils and other analogous affections are treated by him with *sulphuric acid*, which he regards as almost a specific. He has used the acid with constant success for five-and-twenty years. "As soon as the patient applies for relief," he says, "I put an adult on elixir vitriol, twenty drops three times a day, in a glass of sweetened water, one hour before meals, previously smearing the teeth well with fresh butter or chewing a piece of fat pork, for a sure protection to the teeth. This is much better than sucking through a quill, as there is in this way regurgitation enough to throw the acid forward on to the teeth.—Using the butter or pork is a perfect protection, if the teeth are subsequently washed with a solution of bicarb. soda, a heaping teaspoonful to a glass of water. In the use of sulphuric acid in this way, the boil (or crop) then on hand will soon melt away, and there will be but one effort more to return before they will finally disappear, no more to reappear. The acid should be kept up in ten-drop doses for at least two weeks after the boils have disappeared. To assist in their local treatment, to effect a speedy cure and afford relief from pain and soreness, I apply a piece of common adhesive plaster, cut round, sufficiently large to cover the tumor to the extent of the areola, clipping the edges so that it will set smooth; or a little shoemaker's wax spread on a cloth will do just as well. I have made this application to saddle boils, and next day rode in the saddle very comfortably, the boil progressing to maturity with great rapidity, with very little pain, and sometimes effecting an abortion at once.

"In this connection, I have read in your *Periscope* a very interesting and scientific article on the sulphides of soda, potash, and calcium, as an antidote for all these ills of humanity. And now, right here let me suggest, perhaps the chemical play of affinities in *Nature's* chemical laboratory may evolve the sulphides in the same way that chloral *amateurs* claim that hydrate chloral is metamorphosed in the blood to chloroform."—*Druggists' Circular & Chemical Gazette.*

THE *Revue Industrielle* states that apples may be preserved in perfect condition by packing them in dry plaster.

*Syphilitic Lichen.*—The case was exceedingly well marked, and was at the forty-third day from the exposure.

The treatment which the patient was under was as follows:

Good food and porter; vapor bath not lasting more than ten minutes, once or twice a day; and the following drugs: ℞. Potassio tartrate of iron, ʒ vi.; aqua, ℥ viii. M. Teaspoonful of this mixture with from $\frac{1}{8}$ to $\frac{1}{16}$ of a grain of bichloride of mercury, t. i. d.

The mercury is combined, not for the purpose of producing its specific effect, but simply for its tonic and alterative influence. Salivation is not desirable.

In connection with the above, the patient was also receiving Fowler's solution, gtt. xxv. t. i. d., administered in milk and after meals.

The arsenic was commenced in doses of five or six drops, and increased to ten as soon as possible, and from that the remedy was to be carried on until *some nausea* was produced. The twenty-five drops were being taken without inconvenience.

The idea entertained was, that arsenic is not specially serviceable in the treatment of this class of affections, unless carried up to the point of producing some nausea. Prognosis guarded with regard to perfect cure in this case.—*N. O. Med. and Surg. Jour.*

*The Local Application of Bromine in the Treatment of Carcinoma of the Cervix.*—Henneberg has found that the alcoholic solution of bromine, when used in the proportion of one to five, is a useful application in cancer of the neck, both in the treatment of the open wounds produced by the exfoliation of the growth, and also when injected into the substance of the diseased tissues. This is essentially the plan of Schroeder. Under the guidance of Steck, the author performed some experiments to decide what the action of this solution actually was upon growths that had been removed. He obtained portions of a typical case of schirrhus and placed them in the bromine solution for forty-eight hours. They were then removed, and on examination seemed to consist merely of traces of connective-tissue with spindle cells. In another case, the cancerous masses were observed to almost wholly disappear under the action of the solution.—*Allg. Wien. Med. Ztg.*, 1874.

*Bloodless Operations.*—The paper of the evening (which we publish in full elsewhere in this journal) was read by the President, on Esmarch's method of applying the elastic bandage.

Dr. James R. Wood said he had operated on twenty-five cases, aided by the bandage, and had not noticed any evil effects. He considered it a marked improvement in surgery, but thought the rope a disadvantage, and for this purpose substituted the bandage doubled on itself.

Dr. Frank H. Hamilton considered it a valuable aid in operating.

Dr. Krackowizer said that he had operated with it for the first time over thirteen months ago.

It was important to use great care in applying it in cases where there was suppuration, so as not to force pus into healthy tissué, and for this reason suppurating surfaces should be bridged over so as not to allow such an accident to take place. Much injury might result from excessive constriction of the band, and for this reason the softer it was the better.

Esmarch has devised recently a clamp to secure the ends of the rope, but Dr. Krackowizer did not think it was necessary, inasmuch as a tape tied round them was more convenient. He was of opinion that the webbing might act as a vehicle of septic matter, and for this reason he had never used it, substituting rubber hose slit up through the middle.—*N. Y. Med. Journal.*

*Silicate of Soda in Gonorrhœa.*—Prof. Sée, of Paris, recommends this treatment. His observations extended over a period of six weeks, and were mainly made upon two classes of patients, those with acute or chronic blenorrhagia, with or without orchitis, and those with ulcers of the penis, sometimes phagedenic and complicated with inflammatory phymosis. The solution generally used was three grammes (grs. 46) of the silicate of soda in one hundred grammes (about ℥iv.) of distilled water, the strength of the solution being varied according to the extent of the inflammatory phenomena present in each case. Sée says that the silicate of soda can be recommended in these affections in consequence of its cheapness, the ease and freedom from risk with which it may be used, its safety, and the promptness of its action.—*Med and Surg. Rep.*

*Ergotin in Croupous Pneumonia.*—Dr. Wycisk, acting on the principle that this drug contracts the vessels and so prevents exudation from them, has treated six cases of croupous pneumonia in this way, and gives the following report: In one such case, marked by an excessive highly albuminous expectoration, it ceased entirely two hours after the administration of the drug in powders of nine grains each every quarter of an hour. The abundant râles in both lungs soon diminished, so that there was only a slight crepitation at the original focus of the disease. These good effects lasted for two days. Two relapses occurred, however, but the same good effect was again obtained by the administration of the ergot. After the second relapse, ten-drop doses of the tincture of ergot were given four times daily, until convalescence was fully established. In the five other cases the ergot was used early, and none ended fatally, none became chronic, and none left appreciable deposits behind them; in all of them the exudation was decidedly checked by the ergot. It is, however, held to be a dangerous remedy when the lungs are considerably infiltrated, where there is emphysema, where the cerebral arteries are weak, and where the patients are feeble and decrepid.—*Allg. Med. Central-Ztg.*, No. 88.

*Otto of Roses.*—The *Moniteur Industrial Belge*, in an interesting article on this costly perfume, says that the manufacture is largely carried on in the valley of Kisanlik, Roumella, the annual production of the rose farms of which amount to 4,400 pounds of the otto per year. As it requires about 130,000 roses, weighing some 57 pounds, to make an ounce of the oil, some idea of the extent of the plantations may be formed from the above given total.

The flowers are gathered in the middle of May, and the harvest continues for three weeks. The blossoms collected each day are at once worked, in order that none of the odor may be lost. The process consists in distilling them in water, and then causing the water alone to undergo distillation, when the oil is skimmed from the surface. The labor is principally done by women and children, at wages of about ten cents per day.

The otto is always adulterated before transmission to market, with one-third or one-fifth its quantity of geranium oil.—*Druggists' Circular and Chemical Gazette.*

*Modern Medical Treatment.*—An editorial article in a British cotemporary closes with the following thoughtful words: Our treatment has assumed a character too decidedly stimulant, and not quite sufficiently nutritive. Stimulants ought to be regarded as auxiliaries to nutrition more than they are at present. Nutritive material, as milk, meat-juice, eggs, and various forms of starch, ought to form a greater matter in the dietary of the sick than stimulants, whether nitrogenized or alcoholic; such materials, when assimilated, give supplies of force. Stimulants may assist in their assimilation, and do so; but in themselves, stimulants only furnish limited supplies of force-bearing material. They are, however, a means by which the system may reach some of its physiological reserve fund. Such use may be advantageous or pernicious, according to circumstances; and an ill-regulated or excessive process of stimulation may give results as disastrous, as a wise and intelligent resort to stimulants may be beneficial in its consequences.—*Philadelphia Med. and Surg. Reporter.*

*Ice in Diphtheria.*—This is no novelty in therapeutics, but it has lately been urged with new vigor by Dr. Meyer, in the *Jahrbuch für Kinder Krankheiten.* Even in children under one year he directs small pieces of ice to be put frequently into the mouth, followed, if possible, every minute or two by a teaspoonful of iced water. The ice must be pure, and therefore all artificially prepared is best. In severe cases the external use of cold, by means of an ice-bag applied round the throat, is very useful. The author has found that by this mode of treatment the fever soon diminishes, and the diphtheritic membrane is detached and expectorated. It is only in exceptional cases that the disease extends nevertheless to the larynx.—*Med. and Surg. Reporter.*

*Chronic Orchitis.*—When the patient was admitted, the testicle was very much enlarged, and exceedingly hard.

A local application of hydrochlorate of ammonia, of the strength of an ounce and a half to the pint of water, had been kept continually applied, and the testicle at the end of a week or ten days was quite soft and natural to the touch. It was next ordered to be strapped.—*N. O. Med. and Surg. Jour.*

*The Treatment of Ammoniacal Cystitis by Benzoic Acid.*—In a second paper (*Archives Generales de Medecine*) MM. Gosselin and Robin advise the use of benzoic acid as a corrective to the ammoniacal state of the urine. The drug may be given dissolved in large quantaties of water, or suspended in mucilage. The initial dose is 15 grains, but it should be rapidly increased to 45 or 60 grains, and may even be carried in some cases to 90 grains; at this dose, however, heat and dryness of the fauces begin to be complained of. The action of the acid upon the altered urine is not immediately recognized; but in seven or eight days the ammoniacal character and fœtidity generally disappears, and phosphatic deposits cease to form. The conclusions of this article are as follows:

1. The ammoniacal state of urine causing a large part of the accidents which follow operations on the urinary organs, it is desirable to diminish or suppress that condition.

2. Benzoic acid, balsams which contain it, and probably other vegetable products (salicine, cinnamic acid, etc.), produce this result.

3. Hippuric acid, which is the product, acts in several ways: (*a*) by forming hippurate of ammonia, which is less tonic than carbonate of ammonia; (*b*) by retarding the decomposition of the urine, and consequently the production of the carbonate of ammonia; (*c*) by preventing the formation of insoluble phosphatic deposits which are the cause of cystitis and the origin of calculus.

4. The administration of benzoic acid is advisable for patients suffering from ammoniaco-purulent cystitis, and particularly for those who aie to undergo operations upon the urinary organs.—*New Remedies.*

*Chlorate of Potash in Ozœna.*—Dr. Eyselein, of Blänkenberg, reports a very obstinate case of ozæna, which he had cured by painting the ulcerous nasal membrane with a solution of chlorate of potash, one part to six of water (by weight).—*Med. and Surg. Reporter.*

*Chloral Subcutaneously in Asthma.*—The hypodermic injection of three grains of chloral, dissolved in twenty minims of water, will relieve in ten minutes a violent asthmatic spasm, according to Surgeon Major Baillie, R. N.—*Med. and Surg. Reporter.*

*On Persistent Hemorrhage.*—I read with considerable interest an article on Hemorrhagic Diathesis, by Dr. Chase, wherein he relates his experience in a case of persistent recurring hemorrhage following extraction of a tooth. I think such cases are far more common than one would think from the meagre notice given to this accident in works on surgery.

It fell to my lot to have an experience almost identical with your correspondent (*Reporter*, Dec. 5, 1874), wherein I used all the approved remedies and a great many expedients of my own suggestion, all followed by mortifying failure. I finally resorted to the actual cautery, using for that purpose the cautery-iron figured in Dr. Thomas' work on "Diseases of Women," used in applying actual cautery to the neck of the womb. It has a round bulb which nicely fits the orifice left by the extraction of a tooth. I think in his case (that of Dr. Chase) had it been used thoroughly, the bleeding would have been checked, as it was in mine, immediately. It is a last resort, requiring some nerve to submit to. It must check the bleeding, because it produces a heavy, thick eschar, impervious to blood, besides coagulating it in the vessels supplying the hemorrhage. Though an old remedy, we should give it a trial in all those cases where life is threatened from the cause under consideration.—*A. Jessup, M. D., in Medical and Surgical Reporter.*

*Night Sweats of Phthisis.*— Sulphate of atropia is regarded as an excellent remedy for their arrest. It is usually administered in a simple dose at bedtime of $\frac{1}{60}$ of a grain.

Aromatic sulphuric acid.

Niemeyer's pill. Niemeyer's pill and atropia used in conjunction. Oxide of zinc and hyoscyamus were said to be efficient with their cases.—*New Orleans Med. and Surg. Journal.*

*Whooping Cough.*—Dr. Wilde (*Deutsches Archiv*) claims that he can cure every case of whooping cough within eight days, by the following mixture:

℞. Chloroformi, f. ℥ i.; æther sulphur, f. ℥ ij.; ol. terebinth, f. ℥ iij. M. is poured on a cloth and held about two inches from the mouth of the patient till the paroxysm subsides.—*Chicago Med. Examiner.*

*The Use of Carbolic Acid in Cheesy Pneumonia.*—Professor Tommasi states (*Il Morgagni*, quoted in *Gazetta delle Cliniche*) that he has found carbolic acid very useful in cases of cheesy pneumonia which have gone on to the formation of pulmonary abscess. The medicine is administered in the form of solution (one part in forty of water, sweetend). In two years he has met with six cases, of which he relates two.

The first case was that of a boy, aged six years, who had hectic, sweats, extreme emaciation, and moderate expectoration of sanguineous fœtid pus. Cinchona, lactate of iron, cod-liver oil, and raw meat produced but little improvement. He now had half a gramme of the carbolic acid solution daily, the dose being increased to a gramme. At the end of a fortnight the fever ceased, the expectoration diminished and became inodorous, and in four months he was quite restored to health.

In the second case, there was abundant expectoration of inodorous pus. The usual restorative means failed; but, after the solution of carbolic acid had been given in daily doses of a gramme to a gramme and a half, the expectoration diminished, and the patient became free from hectic and regained his appetite, and his nutrition was improved. In two months the expectoration had ceased, and a respiratory murmur, though weak, could be heard in the portion of lung which had been hepatized.—*London Med. Times.*

*Catarrh and Paralysis of the Bladder.*—For these complaints, Dr. Willimsky, of Greifswald, in an inaugural thesis, recommends the watery solution of ergot. A dilute solution should be thrown into the bladder, in paralysis, thrice a day; in acute catarrh of the organ, once a day. In either case, the bladder must be thoroughly emptied before the ergot is used. Successful cases are quoted in support of this treatment.—*Med. and Surg. Reporter.*

*Iodide of Potassium in Diphtheria.*—A writer in the *British Medical Journal* says: "In diphtheria, iodide of potassium is looked upon by many practitioners as the best remedy we possess. Here its alterative and sedative actions are laid aside, and we have it doing duty as an antidote to the diphtheritic poison; although, so far as can be seen, it exercises no new influence.—*Med. and Sur. Rep.*

*Boldo.*—Yet another South American plant, with an odd name, applies for admission to the Materia Medica. This time the new comer is introduced by Messrs. Dujardin, Baumetz, and C. L. Verne.

Boldo is a tree found in Chili, of a height of five or six feet, isolated on mountainous regions, with yellow blossom and a verdant foliage. Its barks, leaves, and blossom possess marked aromatic odor, resembling a mixture of turpentine and camphor. The leaves contain largely an essential oil. It contains an alkaloid which is already called "boldine." Its properties are chiefly as a stimulant to digestion, having a marked action on the liver. Its action was discovered rather accidentally, thus: Some sheep which were liver-diseased were confined in an enclosure which happened to have been recently repaired with boldo twigs. The animals eat the leaves and shoots, and were observed to recover speedily. Direct observations prove its action: thus, one gramme of the tincture excites appetite, increases the circulation, and produces symptoms of circulatory excitement, and acts on the urine, which gives out the peculiar odor of boldo.—*New Remedies.*

*Hysterical Spasms.*—Dr. E. Chenery, of Boston, says, in the *Medical and Surgical Journal*, of that city:

"For many years, the writer's panacea in the treatment of hysterical spasms has been the internal use of a mixture of chloroform and laudanum, after the paroxysms have been relaxed by the inhalation of chloroform, so that the patient could swallow. One good dose of this mixture has usually sufficed to explode the fits, and persons who have been frightfully convulsed have in this way been speedily made 'to take up their bed and walk.'"—*Med and Surg. Reporter.*

*Chronic Ulcers.*—In general, these cases are treated by strapping with adhesive plaster. Three or four cases of large ulcers upon the legs, having heavy indurated edges, were doing very nicely under the local influence of tincture of iodine applied to the indurated parts. The ordinary tincture is painted freely over the induration, and then covered with adhesive straps in the usual manner. Dr. Housekeeper instituted the plan of treatment, and had obtained results eminently satisfactory.—*N. O. Med. and Surg. Jour.*

*The Stamp Act on Medicines.*—For the benefit of such of our subscribers as are engaged in the Drug business, we publish the following section of the Internal Revenue Law:

"SECTION 22. That hereafter nothing contained in the Internal Revenue Laws shall be construed so as to authorize the imposition of any stamp-tax upon any medicinal articles prepared by any manufacturing chemist, pharmaceutist or druggist, in accordance with a formula published in any standard Dispensatory or Pharmacopœia in common use by physicians and apothecaries, or in any pharmaceutical journal issued by any incorporated college of pharmacy, when such formula and where found shall be distinctly referred to on the printed label attached to such article, and no proprietary interest therein is claimed. Neither shall any stamp be required when the formula of any medicinal preparation shall be printed on the label attached to such article, where no proprietorship in such preparation shall be claimed."

*Acute Articular Rheumatism.*—The full alkaline treatment is commonly adopted in the treatment of this affection. Dry cotton is the most commonly used local application. Hop poultices are occasionally employed.

If the temperature becomes *high,* demanding active measures for its reduction, the patient is placed in a bath of the temperature of 60° F., and permitted to remain there until the temperature of the body has fallen within reasonable limits. Dover's powder is given to relieve pain, and, as soon as the acute stage has passed, quinine gr. xv. per diem.—*New Orleans Med. & Surg. Journal.*

*Acute Hematuria Treated Successfully by Local Applications.*—Dr. E. L. Keyes (*Archiv. Dermatology*) describes a case of unusual interest. A young, robust man was attacked by hematuria. After a long course of general treatment, without avail, a careful examination was made, proving that the source of the blood was the prostatic sinus. This sinus was cauterized by the application of solid nitrate of silver. In two days the application was renewed. The blood stopped in twenty-four hours. Symptoms of cystitis of the neck came on, but gradually disappeared, and the patient did pass blood with his urine.—*Detroit Rev. of Medicine.*

*A Mode of Reducing Strangulated Hernia.*—In a recent number of *La France Médicale* it is reported that the following method was adopted by Perrin as a novel procedure in the case of an inguino-scrotal hernia, which had become strangulated, and in which serious symptoms had already shown themselves. Taxis had been thoroughly tried under an anæsthetic, but without success. An attendant was therefore directed to take hold of the patient's legs, and placing them on his shoulders to raise him up until he rested only upon the shoulders and head. The body being thus very strongly flexed forwards, the integuments of the abdomen became so relaxed that Perrin was able by manipulation to reduce the hernia to one-half its former volume, by the return of the fluid which the sac had contained, into the peritoneal cavity. The patient was then placed in the horizontal position, and the gut was completely restored. There is hardly any novelty, however, in this operation, for American surgeons have practiced it in repeated instances.—*New York Medical Record.*

*Aromatic Syrup of Senna.*—℞. Alexandria senna, ℥ iv.; jalap, ℥ iss.; rhubarb, ℥ss.; cinnamon and clove, each, ʒ i.; nutmeg, ʒ ss.; oil of lemon, gtt. xx.; sugar, ℥ xxiv.; dilute alcohol, q. s.

Reduce the root, leaves and spices to a moderately fine powder, and treat with the diluted alcohol, by percolation, to exhaustion; when about one quart has passed, evaporate by means of a water bath to eighteen fluid ounces, and filter if necessary. Then add the sugar and dissolve by water bath heat; when cold add the oil of lemon. The product should measure thirty-two fluid ounces.—*Detroit Review of Medicine.*

*Diarrhœa of Phthisis.*—Among the many remedies and combinations of remedies that have been used, the following prescriptions have obtained some reputation for good:

℞. Opium, $\frac{1}{2}$ to 1 grain; bismuth subnit., gr. xv.; ipecac, $\frac{1}{2}$ grain. M. Taken p. r. n.

℞. Nitrate of silver, $\frac{1}{4}$ gr. to $\frac{1}{2}$ gr.; opium, $\frac{1}{2}$ gr. to 1 gr. M. Ft. pil. To be taken p. r. n.

℞. Strychnia sulph., $\frac{1}{24}$ gr.; bismuth subnit., 15 to 20 grs. M. Taken p. r. n.

*The Preservation of Leeches.*—Mr. George H. Burnham, writing to *The Larboratory*, remarks:

The following method for the preservation of leeches has, in my experience, been most successful. Half a dozen "ten-penny" nails are placed at the bottom of a glass jar holding about eight pints—upon them is placed a piece of very porous sponge.

The jar being filled with water, we have an aquarium in which two dozen leeches will live for many months without a single loss by death; a piece of lawn of about one hundred meshes to the square inch should be secured by a strong rubber band to the top of the jar in order to prevent the escape of the leeches.

When the water is changed, the sponge should be thoroughly cleansed, to remove a quantity of slimy matter free from the cells.

It is said that the presence of metallic iron in water prevents it from becoming putrid. This influence is very marked in water in which leeches are preserved, in consequence of which, the change of the water becomes unnecessary except after long intervals.

The jar containing the leeches should be kept at a comparatively even temperature from 55° to 65° F., excluded from sunlight, and a fresh quantity of water added every five or six days.—*New Remedies.*

*Treatment of Pleurisy.*—With an Appendix of Casos, showing the Value of Combinations of Croton Oil, Ether and Iodine as Counter-irritants in other Diseases. By John W. Corson, M. D. Cloth; pp. 31. Wm. Wood & Co., New York.

As a substitute for blisters the author recommends the application of the following, called mild croton oil paint:

℞. Croton oil, 1 part; strong ether, 2 parts; tinct. iodine, 5 parts. Mix. Apply two or three coats at a time with a camel's hair brush, over a small surface once a week. This is useful for children, females, and sensitive males.—*Detroit Rev. of Med.*

*Sprains.*—Foot bath, hot as can be borne, for half an hour, followed immediately by free rubbing with liniment composed of soap liniment with a tmall quantity of acqua ammonia and oil of rosemary, and then a snugly applied bandage.—*New Orleans Med. and Surg. Journal.*

*Rheumatism and Neuralgia.*—Dr. J. E. Jackson finds that the *shortest* cures of rheumatism are by the opium and alkaline treatment, together with *veratrum viride,* in all cases of the *acute* and sub-acute forms where the pulse needs controlling. Morphia, quinine, hyoscyamus, and chloral hydrate in appropriate doses, and according to the part affected, have been his most successful remedies in neuralgia, together with vesication, Granville's lotion, or equal parts of chloral hydrate and camphor combined, and painted or rubbed over the seat of pain. He has prevented the attacks in several cases of recurring hemicrania by using the following prescription:

℞. Liq. potas. arsenit., f. ʒ ij.; Hall's sol. strychniæ, f. ʒ vj.; morph. sulph. gr. ij. M.

Take ten drops three times a day in water after eating. Dr. Senderling has found obstinate cases of sciatica cured or relieved by pil. iodoform et ferri.—*Trans. of the Med. Society of the State of Pennsylvania,* vol. x, pt. i., pp. 243 and 277.

*Phagedena.*—The case was one of sloughing upon the foot, dependent upon syphilis. Bristow's solution of bromine was employed in this case to destroy the phageden, and the applications followed by yeast and charcoal poultice. The solution is made as follows:

℞. Bromine, ʒ i.; bromide of potassium, ℈ ii.; aquæ, ʒ ii. M.

It is claimed for this solution that the appetite is almost invariably improved within twelve or twenty-hours after its application to the affected part. This was said to be the peculiar effect produced by the application.—*New Orleans Med. and Surg. Journal.*

*Treatment of Hemorrhoids by Injections of Ergot.*—Dr. G. W. Semple reports, in the *Virginia Medical Monthly,* five cases of piles, two of which were accompanied by prolapsus of the rectum, which he treated successfully by injecting into the rectum, after every fecal discharge, half a drachm fl. ext. ergot with half an ounce of water. One had a greatly enlarged spleen which was reduced to its normal size by the treatment. A third was that of a pregnant woman who suffered no inconvenience from the treatment.—*St. Louis Med. and Surg. Jour.*

*Preservation of Vegetable Infusion by Chloroform.*—Mr. J. B. Barnes has recently communicated some interesting experiments made in his laboratory upon the perservative effects of chloroform. He finds that infusions of calumba, chiretta, malt, and senna will keep good for a reasonable time by adding five minims of chloroform to every eight fluid ounces; and three minims will suffice to preserve the same quantity of infusion of roses. It will be easy to add chloroform to concentrated infusions, so that when diluted, each sixteen ounces may contain ten minims of chloroform. He also tried the effects upon two samples of mucilage of acacia, in the proportion of one and two minims, respectively, to the fluid ounce; after six weeks both appeared to be as fresh as the day they were made.

The same effect was found to follow a similar treatment of mucilage of tragacanth.

Not only is alcoholic fermentation prevented by this means, but when chloroform is added in sufficient quantity to fresh milk, lactic fermentation is also prevented, twenty minims of chloroform being sufficient to preserve eight fluid ounces of milk although being exposed for five days in a warm place.

Milk, when preserved in this way, needs to be boiled before using in order to drive off the chloroform.—*New Remedies.*

*A Diagnostic Hint in Old Ulcers.*—In a lecture on chronic ulcers of the leg, in the Detroit *Review of Medicine and Pharmacy*, Dr. McGraw says:

"I will give you a rule for diagnosis. It has very few exceptions. Idiopathic ulcers, which occur in the upper third of the leg, are syphilitic. When you are called upon to treat such cases, inquire whether the ulcer originated in an injury; if not, treat the patient for syphilis without further ado."—*Med. and Surg. Rep.*

*Compound Tincture of Opium, (Diarrhœa Mixture; formula of Dr. E. R. Squibb).*—℞. Tincture of opium, f. ℥ i.; tincture of capsicum, f. ℥ i.; spirits of camphor, f. ℥ i.; purified chloroform, f. ℥iij.; stronger alcohol, q. s., ad. f. ℥ v. Mix. Dose, one teaspoonful in water for adults.—*Proceedings Am. Phar. Association.*

*New Process for Rendering Wood Incombustible.*—A Boston clergyman, the Rev. Dr. Jones, has distinguished himself by inventing a process for rendering wood incombustible, for which he obtained a patent. The wood is made at the same time impervious to dry rot and decay, so that two important ends are attained at once. Most of the old methods of preserving wood only render it more liable to fire, as was shown not long ago in the burning of the landing stage at Liverpool. Dr. Jones subjects the wood to a pickling process, in a solution of tungstate of soda and water of the specific gravity of 1.2. The tungstate is made by the addition of tungstate of lime to hydrochloric acid and salt, and it produces in the process as much chloride of lime as will pay all working expenses. The tungstate of soda, from experiments that have been made publicly and privately during the last three years, is proved to render soft woods, such as white and yellow pine, as hard as oak or teak, and it will also restore wood that has been affected by dry rot to the original condition of durability.—*Druggists' Circular.*

*Tonic Prescription.*—The following prescription is regarded specially beneficial as a general tonic in syphilitic patients, and has been found to act exceedingly well in those cases in which the functions of the stomach have been almost destroyed by the use of alcoholics :

℞. Quinia sulph., grs. xxx. ; tr. ferri chlorid, ʒ iii. ; strychnia sulph. gr. $\frac{1}{4}$ ; syr. zingiberis et, aquæ ad., ℥ iii. Teaspoonful every four hours.—*New Orleans Med. and Surg. Journal.*

*Dysentery.*—Dr. Cushing, in the *Chicago Medical Journal,* reports remarkable success in the treatment of dysentery with the following prescription, preceded by a saline cathartic :

℞. Creasote, gtt. xx. ; acetic acid, gtt. xl. ; morphia, gr. ij. ; water, ℥ ij. M. Teaspoonful every two hours until relieved.

*Menorrhagia.* —Dr. Gray, in *American Journal of Insanity,* says the following formula is excellent in "*profuse hemorrhagic menstruation :* ℞. Gallac acid, grs. xx. ; fluid ext. ergot, grs. xx. to xl. ; glycerin, ʒ j. ; water, ℥ iij. M. and administer three times a-day.

*The Action of Morphia Subcutaneously Injected.*—From one thousand experiments made upon himself by Chouppee (*Gazette Méd.* No. 35, 1874) the conclusion was drawn that morphine injected in *loco dolenti* develops its anæsthetic action in two to two-and-a-half minutes earlier than when injected at a distant place. Pain ceased sooner after direct than after general application. A further direct proof of the local anæsthetic action of this agent was derived from the employment of concentrated solutions. While an injection of distilled water and a weak solution of morphia (1-150) caused sharp pains at the point of insertion, strong solutions (1-50 or 1-129) cause no perceptible pains. The indication, therefore, is to make the injections at the seat of the pain and to use concentrated solutions.—*Allgem. Wiener Med. Zeit.*

*Treatment of Typhoid Fever by Air Baths.*—M. Miramont de Mery (Savoy) suggests, as a means of reducing temperature, air instead of water baths. The patient is placed on a hair mattress, covered only by a sheet, and in the middle of a large room. The bed should be changed several times a day, and in the event of delirium or exacerbation of febrile symptoms, the patient is to be made to walk about in his shirt till he is cold, when consciousness will return.—*Jour. de Med.—Clinic.*

*Arsenic in Asthma.* — Dr. C. Paul (*American Journal Medical Sciences*) reports several cases of spasmodic asthma which were greatly benefited by the administration of arsenic. He says the remedy must be persevered in until constitutional effects are produced, before any benefit can be expected ; it should be thoroughly tried. It has succeeded after all other drugs failed. He gives Fowler's solution in from ten to fifteen minim doses, after meals, and in some cases he uses it hypodermically.—*Canada Lancet.*

*Sulpho-Carbolate of Zinc in Pruritis.*—Mrs. J. G. Brown, M. D., of the Illinois Women's Hospital, (*Medical Examiner*) recommends as an effectual remedy for obstinate pruritis of the vulva, a solution of sulpho-carbolate of zinc, 30 grains to the ounce of water. After washing with warm water, the solution is applied and left to dry. The application may be made twice a day, to begin with, afterwards once a day, or two or three times a week.—*Canada Lancet.*

*The Artichoke in Rheumatism.*—Dr. Copeman, in the *British Medical Journal,* recommends a tincture of the leaves of the artichoke, *cynara,* gathered while they are full of juice, just before the top of the vegetable is fit for food.

He says that, in his experience, no other medicine has appeared to be so efficacious in rheumatism as cynara, whether in the acute or chronic stage. Dr. Copeman usually administers it in the following form :

℞. Potassa bicarb., ʒj. ; aquæ camph. ad., ʒ viij. ; tincture cynaræ, ℥j. ; syrupi papaveris alb., ℥ss. M. Two tablespoonsful to be taken every four hours, with as much of the extract of cynara as would make two moderately sized pills. Lemonade is given to quench the thirst.—*Med. and Sur. Reporter.*

*Treatment of Spasmodic Asthma.*—Dr. Julio J. Lamadrid recommends the combination of chloral hydrate with the bromide of potassium in the treatment of spasmodic asthma. The following is the formula which he employs :

℞. Chloral hydrat, ʒj. ; potassi bromide, ʒ ijss. ; syr. flor. aurantii, aquæ dest, aa. f. ℥ i. Sig. A teaspoonful in half a wine-glass of water every two hours, until sleep is induced or dyspnœa is relieved.—*Philadelphia Medical Times.*

*Gonorrhœa.*—Dr. Haberkorn, of Berlin, reports excellent results in both acute and chronic gonorrhœa, with the following injection : ℞. Quiniæ sulphatis, grs. vj. ; glycerinæ, ʒ ij. ; aquæ, ʒ vj. ; acid sulph. dilut., gtts. v. M. Sig. Use about a teaspoonful or two as an injection three times a day.—*Med. and Surg. Reporter.*

*Indurated Bubo.*—Cover the part freely with mercurial ointment, and keep up constant pressure by means of a *hot brick.* Better results were claimed for this method of treatment than any other that had been adopted.

*Mammary Abscesses.*—Quinine in full doses soon as chill occurs. Cease nursing at once, and remove the milk by hand-rubbing, coving the parts with warm lard, and rubbing from the base of the gland towards the nipple.—*Medical Record.*

